# A 10-Year Journey to Diagnosis With Endometriosis: An Autobiographical Case Report

**DOI:** 10.7759/cureus.21329

**Published:** 2022-01-17

**Authors:** Lexi R Frankel

**Affiliations:** 1 Obstetrics and Gynecology, Nova Southeastern University Dr. Kiran C. Patel College of Allopathic Medicine, Davie, USA

**Keywords:** endometriosis of the diaphragm, heavy menstrual bleeding, leuprolide, endometriosis, chronic pelvic pain, endometriosis staging, endometrioma, diagnostic delay, autobiographical case report, laparoscopic surgery for endometriosis

## Abstract

Endometriosis is a multifocal, chronic disease defined by extrauterine endometrial glands and stroma. This case report describes the author’s experience of living with stage IV endometriosis, including a 10-year diagnostic delay, the impact on daily life, management, and treatment. The diagnostic delay for endometriosis averages between seven to nine years globally, which imparts significant physical, psychological, and financial effects on the lives of patients.

## Introduction

Endometriosis is defined by the presence of extrauterine endometrial glands and stroma [1]. It is a multifocal chronic disease without known cure or prevention [2]. Most commonly, endometriosis is found in the pelvic cavity, ovaries, uterosacral ligaments, and rectouterine pouch [3]. More rarely, endometriosis can be found in the gastrointestinal or urinary tracts, pleura and pericardium, heart, lung, brain, umbilicus, inguinal area, breast, pelvic nerves, and surgical scars [2]. It has even been described in rare cases in males [4].

The most frequently presenting symptoms of endometriosis include dysmenorrhea, menstrual pelvic pain, dyspareunia, infertility, and pelvic mass [5]. This disease, however, can manifest with a wide range of less common symptoms including dysuria, dyschezia, back or thigh pain, intermenstrual pelvic pain, chest pain, shortness of breath, flu-like symptoms, and migraine [6-8].

Between 1973 and 2021, there have been 22 published endometriosis classification, staging, and reporting systems [9]. The most widely used system, the revised American Society for Reproductive Medicine (rASRM) score, assigns values to endometriosis lesions with points given corresponding to lesion size and presence and location of adhesions. Stage I is minimal with one to five points, stage II is mild with six to 15 points, stage III is moderate with 16-40 points, and stage IV is severe with >40 points [10]. However, even some of the most widely used classification systems have major drawbacks. The rASRM system does not correlate well with pain, fertility outcomes, or prognosis, while another widely used classification method, the Enzian system, does not correlate well with symptoms or infertility [9]. The lack of a gold standard staging system interferes with communication among physicians and patients and makes standardization of optimal treatment near impossible [11]. Most recently in 2021, the American Academy of Gynecologic Laparoscopists (AAGL) announced a new system that is user-friendly, merges previous classification systems, and objectively classifies surgical findings. While the AAGL system discriminates surgical complexity levels better than previous systems, it still does not fully communicate level of disease as it does not include extra-abdominal lesions in classification levels [12].

Endometriosis affects 10-15% of all reproductive-aged women and often imparts physical, psychological, social, and financial effects [7,13,14]. As symptoms of endometriosis are often nonspecific and definitive diagnosis requires laparoscopy, there are often diagnostic delays that have been associated with increased pre-diagnosis endometriosis-related symptoms and higher pre-diagnosis healthcare utilization and costs [14].

This case report describes the author’s experience of living with stage IV endometriosis, including the 10-year diagnostic delay, impact on daily life, management, and treatment. The goal of writing this case report is to provide a resource for my colleagues regarding the variable presentation of endometriosis, its association with lengthy diagnostic delays, and to increase awareness of this condition.

## Case presentation

At age 15, I began experiencing heavy menstrual bleeding, severe menstrual cramping, and dyschezia with menstruation. Between 2010-2020 (ages 15-25), I was seen for these symptoms by three pediatricians, a hematologist-oncologist, a gastroenterologist, a primary care physician (PCP), and four obstetrician-gynecologists (OB/GYNs). Between ages 15-19, I was treated with a variety of progesterone-only oral contraceptive pills (OCPs) secondary to a history of migraine with aura and an etonogestrel implant, none of which adequately controlled pain or bleeding. I was even trialed with a low-dose estrogen-progesterone combination pill, which was stopped secondary to migraine flare. I was instructed to stop all OCPs after these trials, as I was having headaches and nausea without symptomatic relief. I was instructed to take non-steroidal anti-inflammatory drugs (NSAIDS) during menstruation for pain. Until 2020, the only diagnosis I received was “heavy menstrual bleeding,” but was told my symptoms were normal. In 2014, an episode of syncope led me to seek further evaluation. I had iron-deficiency anemia with a hemoglobin of 8.0 and was started on oral iron. I was tested for a panel of bleeding disorders, all of which were negative. In 2016, I became suspicious of whether I had endometriosis and saw two OB/GYNs for evaluation, both of whom agreed the diagnosis was highly unlikely and diagnostic laparoscopy would be not be indicated.

**Figure 1:**
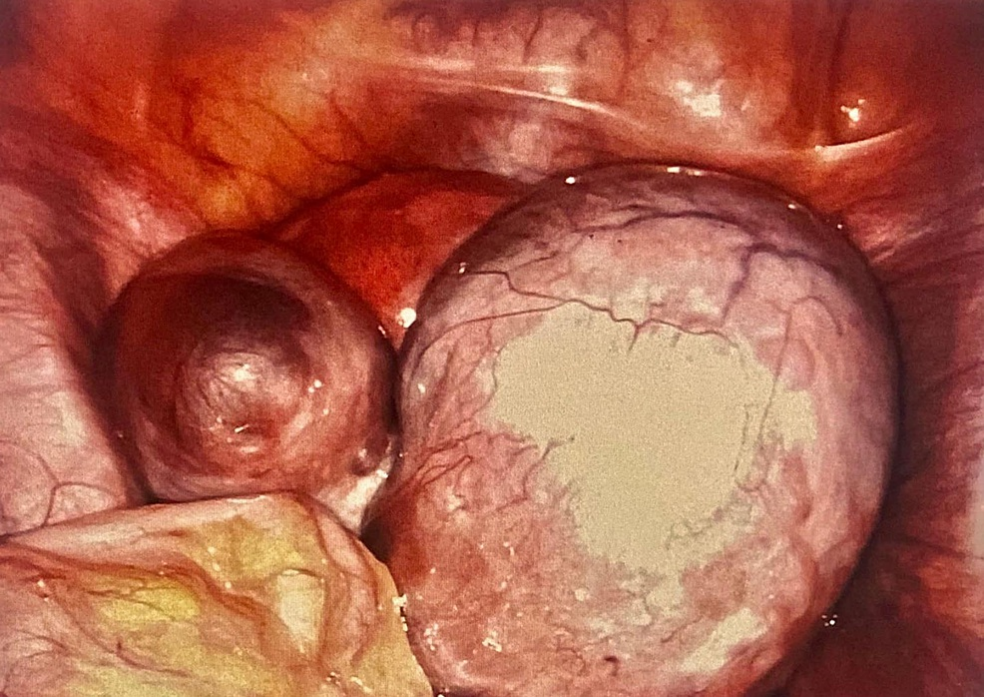
Right ovarian endometrioma (10 cm) and three left ovarian endometriomas, each <5 cm in size.

**Figure 2:**
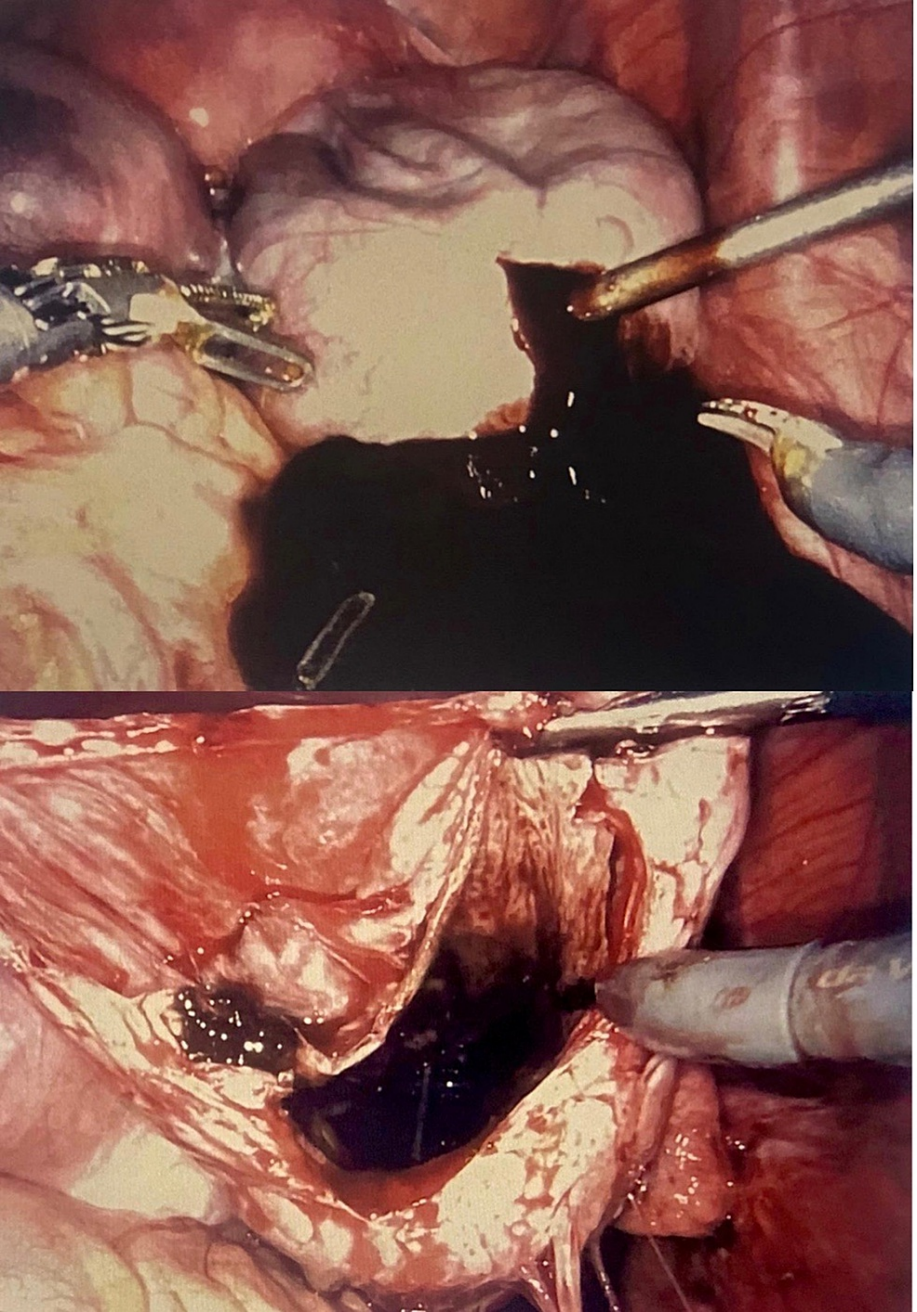
Laparoscopic drainage of right ovarian endometrioma revealing chocolate-like fluid.

**Figure 3:**
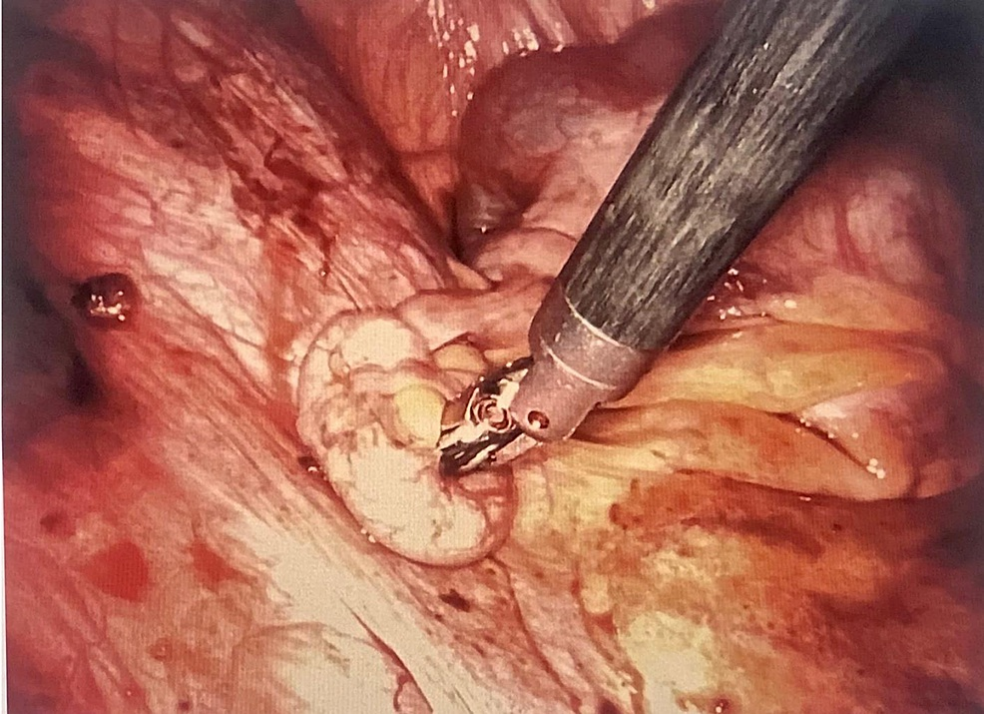
Laparoscopic image showing extensive endometriotic implants on the pelvic wall, ovarian ligament, and uterosacral ligament.

**Figure 4:**
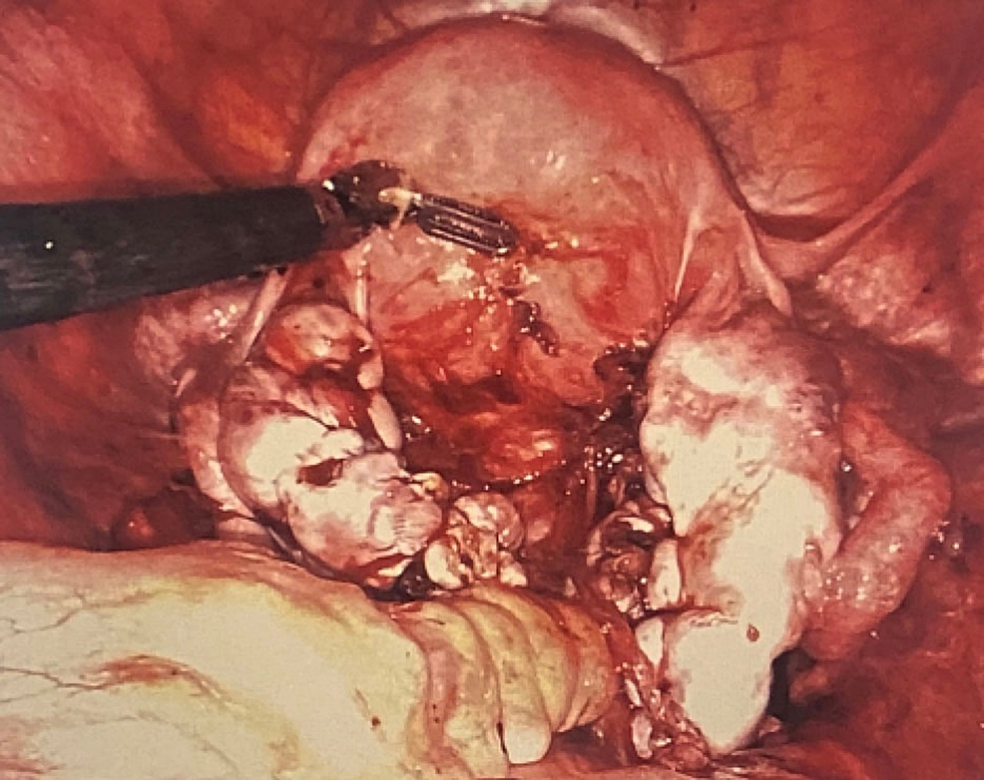
Bilateral ovaries and uterus immediately s/p ovarian endometrioma cystectomy and repair. s/p: status post

**Figure 5:**
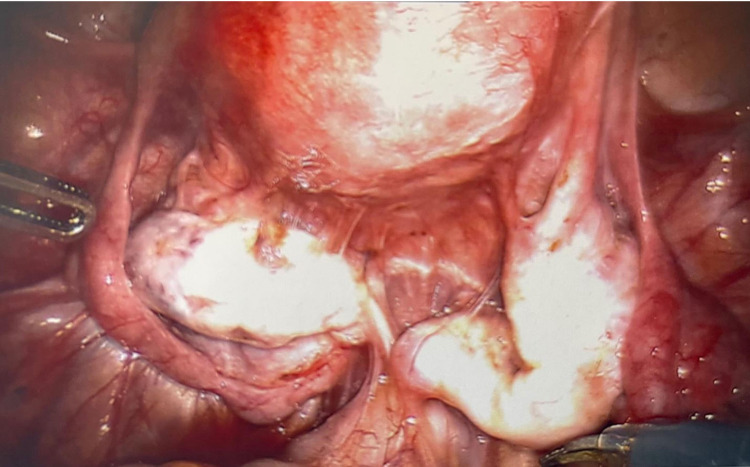
Bilateral ovaries and uterus six months s/p ovarian endometrioma cystectomy. Diffuse adhesions present. s/p: status post

**Figure 6:**
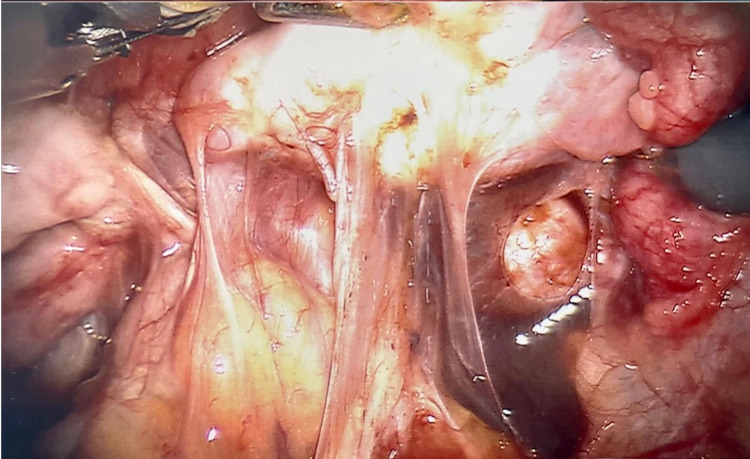
Pelvic cavity six months s/p ovarian endometrioma cystectomy showing diffuse fibrosis and adhesions. s/p: status post

From 2016 to 2020, I continued to treat my pelvic pain with NSAIDs and heat packs. In 2020, while in my second year of medical school, my symptoms rapidly progressed. My pelvic pain increased dramatically and was now more diffuse and intermenstrual including my lower back and thighs. I also began experiencing intermittent constipation and diarrhea with rectal bleeding. Shortly thereafter, a lower abdominal mass became palpable and I sought evaluation by a PCP, who diagnosed me with irritable bowel syndrome and prescribed peppermint oil. I saw a gastroenterologist a few days later, who ordered a pelvic ultrasound (USG) as he presumed my symptoms were more gynecological rather than solely gastrointestinal. The pelvic USG showed complex cystic masses on both ovaries and follow-up was suggested to rule out neoplasm. I was subsequently seen by an OB/GYN and underwent exploratory laparoscopy and cystectomy of bilateral endometriomas (Figures 1-2) and was diagnosed with stage IV endometriosis. Endometriotic tissue was found on the anterior abdominal wall, bilateral ovaries, vaginal wall, appendix, bladder, bowel, rectum, and diaphragm (Figure 3). The ovaries were repaired with good anastomosis and hemostasis (Figure 4). I was subsequently treated with leuprolide and progesterone for a year before repeat-exploratory robotic laparoscopy with endometriotic implant excision/ablation and appendectomy (Figure 5). During this surgery, extensive adhesions and fibrosis of the pelvic cavity and appendix were noted (Figure 6) and chromotubation showed a left fallopian tube hydrosalpinx.

Impact on daily life

Endometriosis is a disease with a highly variable presentation. During adolescence, I would bleed through about two pads per hour during the first couple of days of my cycle. Subsequently, there were many occasions on which I stayed back during social gatherings due to pain and fear of bleeding through clothing. During travel, I always bring more pads and tampons than I expect to use and pack extra medication and fluids.

The nonspecific and extremely difficult-to-diagnose nature of endometriosis has been, in retrospect, one of the most challenging aspects of my disease. I could move forward and manage my symptoms once I knew the cause, but the uncertainty was by far the worst part. Owing to pain, frequent appointments, and surgeries, my endometriosis of course had a large impact on my quality of life, time for family/friends, and even my studies. Now that I have a diagnosis, my disease has less control over my life. I am able to symptomatically manage, plan for menstruation, and treat my symptoms prophylactically.

There is a large financial burden that comes with endometriosis, especially with a diagnostic delay. The costs include the basics from more than the usual number of pads/tampons and heat packs to over-the-counter NSAIDs, prescribed OCPs and other birth control methods, leuprolide, surgical treatment, regular office visits, and imaging. Even after the diagnosis and surgical treatment, the costs continue. I required a dual-energy x-ray absorptiometry scan to evaluate bone mass after leuprolide treatment, ultrasounds every four months, and will eventually need a colonoscopy to evaluate colonic diameter and endometriotic invasion. In the future, my financial responsibility will additionally increase with further surgical and medical treatment.

Managing symptoms 

With menstruation, I experience severe pelvic cramping, dyschezia, and pain with urination. Over the years, I have found that naproxen sodium alternating with Tylenol every four hours provides the best pain control. The naproxen also decreases the quantity and duration of bleeding. I use a microwavable heat pack, which I find very useful for diminishing cramps. If I have work, I often bring transportable hand warmers, which fit discreetly into a sweater or scrub pocket for on-the-go pain relief.

My pain flares are usually spontaneous and unpredictable; however, I have been able to identify a few triggers. Excess caffeine and any amount of alcohol usually cause a flare, so I mostly avoid these. Recent literature has shown that both caffeine and alcohol increase the bioavailability of estrogen, which is detrimental for an estrogen-dependent disease [15].

Over the years, I have learned to identify a cyst rupture. My symptoms include sudden onset unilateral pelvic pain followed by a few days of more diffuse pelvic pain, lower back pain, upper thigh pain, and nausea. I manage these symptoms with ibuprofen or ketorolac with ondansetron as needed for nausea.

I was on leuprolide therapy for a year, which consisted of monthly injections combined with progesterone oral pills. I experienced frequent hot flashes, dizziness, night sweats, mood swings, and headaches. These symptoms were exacerbated by lack of sleep, hot weather, caffeine, alcohol, and dehydration. I found the most relief by cutting out alcohol completely and only having half a cup of coffee per day. I also stayed well-hydrated and brought electrolyte-replacement sports drinks and protein bars with me during outings or during rotations in the hospital. Post-leuprolide therapy, my pelvic pain and other symptoms have decreased dramatically.

Since age 17-18, I have had hiccups and shoulder pain with menstruation almost every cycle. In 2020, the diagnosis of diaphragmatic endometriosis confirmed this symptom’s etiology. The endometriotic implants bleed during menstruation, causing diaphragmatic irritation and spasm. Besides drinking water, I have not had much success with any interventions to alleviate hiccups. Surgical intervention on my diaphragm was foregone for now as the symptoms are not interfering much with daily life and diaphragmatic surgery is not without risks.

Fertility 

Endometriosis of any stage is known to decrease fertility. While it is difficult to predict a woman’s fertility, I was told by my OB/GYN that my fertile age was likely about 10-15 years older than the average woman my age. This determination was made by taking into account the very large bilateral endometriomas and ovarian surgery, which likely decreased my ovarian reserve, my left hydrosalpinx, and stage IV endometriosis. My risk of ectopic pregnancy is significantly increased given the left hydrosalpinx. Endometriosis has shortened my possible window for pregnancy owing to my older fertile age and requirement for eventual treatment with hysterectomy.

Urinary and bowel issues

From 2010 to 2020, I had intermittent pelvic pain with urination. As a result of endometriomas adhered to my bladder, I had intermittent urinary incontinence for a month before the bilateral ovarian cystectomy. For a few months prior to surgery, I also developed urinary hesitancy and had to lift upward on my lower abdomen while urinating to alleviate the pressure my endometriomas were placing on my bladder. The urinary incontinence and hesitancy resolved status post laparoscopy and ovarian cystectomy. Following ovarian cystectomy, I still had pelvic pain with urination, although mostly with menstruation. This improved after the second laparoscopy with bladder implant excision and ablation and post-leuprolide therapy.

In 2018, I began experiencing constipation, which I self-treated with fiber supplements and polyethylene glycol. These symptoms persisted until 2020, when I also developed severe dyschezia, rectal bleeding, and mixed diarrhea and constipation (pre-diagnosis of endometriosis). Post-robotic laparoscopies and leuprolide, my gastrointestinal symptoms improved significantly. Now, fiber and polyethylene glycol are only used as needed for control of constipation and diarrhea with good response. Colonoscopy will be obtained within the next couple years to evaluate colonic diameter; it was, however, deferred for now secondary to adequate symptomatic control. I usually take polyethylene glycol a few days before expected menstruation to prevent constipation and pelvic pain. I have found papaya enzyme supplements taken after meals useful for preventing gastrointestinal upset during menstruation. I also increase my consumption of orange foods, including ripe papaya, citrus fruits, and carrots, which has been studied to decrease bloating and inflammation and to reduce the risk of endometriosis [16].

## Discussion

This autobiographical case report details many common features of endometriosis including chronic pelvic pain, dyspareunia, heavy menstrual bleeding, and dyschezia while also discussing the more uncommon symptoms of endometriosis, including dysuria and hiccups. It additionally describes the complications associated with diagnostic delay and how they affect the quality of life and contribute to the financial burden.

The diagnostic delay of endometriosis averages between seven to nine years globally [17]. In my case, it took 10 years, 10 physicians, 50+ office visits, and a surgery to be diagnosed with endometriosis. The cause of this extensive delay is multifactorial and partly rooted in societal norms, expectations, and taboos. Both patients and providers normalize symptoms, symptoms are suppressed by hormonal treatments, and inadequate diagnosed methods are used [18]. I sought extensive medical care after menarche, but after being told by multiple providers that my symptoms were normal, I stopped asking about endometriosis for several years. It was not until medical school when my symptoms became unbearable and I felt a pelvic mass that I again sought medical attention. Unfortunately, the literature shows that my case was not an outlier; in fact, many women are only diagnosed when their symptoms progress to an intolerable severity [18]. By this point, fertility, quality of life, and costs of healthcare are often greatly affected. I will never know how my endometriosis could have been controlled had it been diagnosed sooner, and this is something that continues to both haunt me and drive my passion for medicine. It is too soon to know how my fertility has been affected or if pregnancy will ever be an option for me. One of the social aspects of endometriosis arises here; other people frequently ask about pregnancy plans and fertility without realizing the 10-year past they have inquired about.

In this case report, I discussed how when I was seen by a PCP, I was initially diagnosed with IBS before my endometriosis diagnosis. It is important to note that while the incidence of IBS is increased in patients with endometriosis, other potential differential diagnoses should not be excluded including deep infiltrating endometriosis of the colon, endometriomas, and other ovarian cysts [19]. The diagnosis of endometriosis becomes muddy in this light as well. Endometriosis is so often associated with many other diseases, such as IBS, overactive bladder, and migraine, these other diseases are diagnosed years before the endometriosis, further contributing to the diagnostic delay.

In the United States, approximately 12% of women with endometriosis will require treatment with hysterectomy [20]. Hysterectomy with or without bilateral salpingo-oophorectomy is commonly the definitive treatment for advanced endometriosis as it significantly reduces pain and recurrence in most cases. However, pre-menopausal hysterectomy has also been associated with cardiac disease, osteoporosis, dementia, and certain cancers [20]. While I have had endometriosis for over 12 years now, I have only been diagnosed for less than two. Although hysterectomy will be my eventual treatment, it is important to delay this surgery as long as possible in an attempt to maintain fertility and pro-estrogenic bodily effects. While it is unclear to what degree my diagnostic delay affected my treatment, it is possible my diagnostic delay was to blame for the necessity of hysterectomy and extensive surgeries.

A limitation of this case report is that I am just one woman. While I have adapted several tips and tricks to manage my symptoms over the years, it is unclear whether they will be successful for others. Although I have endometriosis on several organs, there are countless types and locations of implants about the characteristics or symptoms of which I am unable to speak, given my experiences. Additionally, endometriosis, even of the same location and stage, is experienced differently by each person and staging does not correlate with symptoms or severity [9].

While endometriosis treatment has improved over the past twelve years of my disease, there is a multitude of areas requiring further attention and research in both the medical and societal realms. More efforts should be made to normalize discussing menstruation, especially during adolescence. Menstruating women need to feel comfortable enough to discuss their symptoms with physicians who will listen, inquire, and refer to more experienced providers if necessary. On the research front, we need to be working on reducing the diagnostic delay. More funding should be awarded to the research of diagnostic tests not requiring laparoscopy and to more accurately quantifying the financial effects of this diagnostic delay on patients and healthcare systems.

For me, it was a huge relief to be diagnosed with endometriosis. It allowed me to begin treatment, plan for my future as much as possible, and most importantly, accept my symptoms. My symptoms have had a less negative effect on my quality of life since diagnosis because not only am I on a better treatment regimen, but I am also more open to talking about my experiences. I live a fulfilling life with more passion for medicine because of my disease and while I question some of the decisions that were made during my treatment, I am eternally thankful for my diagnosis and have hope that endometriosis care is evolving (slowly) for the better.

## Conclusions

Endometriosis continues to be a highly enigmatic disease surrounded by controversy and debate. Its extensive average diagnostic delay contributes significantly to quality of life and further research should be conducted to understand and reduce it. Diagnosis is vital to improve treatment and patient acceptance of endometriosis and more efforts should be enacted to improve endometriosis research, education, and awareness.
